# Burden of Pulmonary Hypertension in End-Stage Renal Disease Patients Receiving Maintenance Hemodialysis: Evaluation Factors and Treatment Strategies

**DOI:** 10.7759/cureus.69123

**Published:** 2024-09-10

**Authors:** Karthikeyan Gurusamy, Ramasubramanian V, Shankar P, Kannan Bhaba Velu, Senthilkumar PK, Murugesh Anand, Praveena Daya A

**Affiliations:** 1 Nephrology, Tirunelveli Medical College and Hospital, Tirunelveli, IND; 2 Community and Family Medicine, All India Institutes of Medical Sciences, Madurai, Madurai, IND

**Keywords:** cardiovascular complications, chronic kidney disease, maintenance hemodialysis, pulmonary hypertension, pulmonary hypertension treatment

## Abstract

Background

Chronic kidney disease (CKD) is an emerging public health problem in India. Pulmonary hypertension (PH) is an overlooked cardiovascular complication of CKD. This study aimed to estimate the burden of PH among CKD patients undergoing hemodialysis in a selected tertiary care hospital. In addition, it aimed to determine the various factors associated with PH and response to various treatment modalities.

Methodology

This hospital-based, analytical, cross-sectional study was conducted in the nephrology department of a medical college hospital in Tamil Nadu from March 2023 to March 2024. The study included 150 adults diagnosed with stage 5 CKD and registered for hemodialysis.

Results

Of the 150 participants, 96 (64%) had PH. Of these, 81 (84%) were males and 15 (16%) were females. In the PH group, 58 (60.4%) had type 2 diabetes mellitus, 52 (54%) had been on dialysis for 6-12 months, 67 (69.8%) had chronic glomerulonephritis, 25 (26.04%) had chronic tubular interstitial nephritis, and 72 (75%) had microcytic anemia. PH patients had significantly higher rates of protein-energy malnutrition (26 (48.2%) vs. 67 (80.7%), p < 0.009) and more frequent hospitalizations (19 (35%) vs. 69 (71.9%)) compared to those without PH. Additionally, PH patients exhibited higher incidences of left ventricular hypertrophy (72% vs. 92%), pericardial effusion (0% vs. 65%), and tricuspid regurgitation (16% vs. 100%). Patients treated with a combination of isosorbide dinitrate (ISDN) and hydralazine; extended hemodialysis; and ISDN, hydralazine, and sildenafil showed significant improvement compared to other treatment modalities.

Conclusions

The high prevalence of PH among CKD patients undergoing hemodialysis underscores the importance of vigilant monitoring and targeted interventions.

## Introduction

Chronic kidney disease (CKD) is an emerging public health problem in India. Cardiovascular disease is the most common cause of morbidity and mortality in CKD [1]. Pulmonary hypertension (PH) is a chronic progressive disease characterized by elevated pulmonary arterial pressure. A mean pulmonary arterial pressure (mPAP) greater than 25 mmHg at rest is considered elevated [2]. PH is an overlooked cardiovascular complication of CKD, especially in end‑stage renal disease (ESRD). The prevalence of PH in patients on any kind of dialysis, hemodialysis, and peritoneal dialysis has been reported to be 38%, 40%, and 19%, respectively [3]. PH in patients with ESRD is a serious condition associated with an elevated risk of cardiovascular events and mortality and is an independent predictor of increased mortality in patients with CKD. The pathogenesis of PH in CKD is not fully elucidated. Although identifying PH can be challenging, it is important as management strategies differ for ESRD patients with and without PH. Identifying the presence of PH and the factors associated with it can help in planning appropriate interventions at an earlier stage to reduce mortality.

The risk factors and causes for PH in hemodialysis patients have not been addressed. It is considered to be due to the interaction of multiple aspects of altered cardiovascular physiology. Myocardial dysfunction leading to elevated left ventricular filling pressure and pulmonary venous hypertension are suggested as the predominant causes of PH in CKD [4-6]. The other factors implicated include increased cardiac output [2-4], increased pulmonary blood flow due to shunting across arteriovenous fistula volume overload, anemia, exposure to dialysis membranes, endothelial dysfunction leading to pulmonary vasoconstriction, decreased compliance of pulmonary vasculature, vascular calcification and stiffening, increased thromboxane B2, and pro-brain natriuretic peptide [4-6]. The effect of arteriovenous fistula on the development of PH in hemodialysis patients depends on the type of arteriovenous fistula, duration of usage, and blood flow rates across the arteriovenous fistula. This is probably due to increased cardiac output causing elevated pulmonary artery pressure; however, the association is yet to be established. Few studies have identified age, type of arteriovenous fistula, length of hemodialysis, bone mineral abnormalities, fluid overload, and systolic and diastolic dysfunction as risk factors for the development of PH [3,7]. These risk factors vary with each study and definitive factors for the development of PH are yet to be identified. Hence, this study was designed to estimate the burden of PH and the various factors associated with the development of PH among select CKD patients undergoing hemodialysis in a tertiary care hospital.

## Materials and methods

Study design, setting, and duration

This hospital-based, analytical, cross-sectional study was conducted in the nephrology department of a medical college hospital in Tamil Nadu from March 2023 to March 2024. Ethical approval was obtained from the Tirunelveli Medical College Institutional Research Ethics Committee (approval number: 20232692) before the initiation of the study. The study objectives were explained to study participants and written informed consent was obtained.

Study population

Inclusion Criteria

Adults aged ≥18 years of both genders, diagnosed with stage 5 CKD as per the Kidney Disease Improving Global Outcomes (KDIGO) guidelines [8] registered for hemodialysis in the nephrology department of Tirunelveli Medical College Hospital, Tamil Nadu, and who gave informed written consent for participation in the study were included.

Exclusion Criteria

Known cases of PH, known valvular and congenital heart diseases, and chronic obstructive lung diseases were excluded.

Sample size and sampling technique

A total of 150 adult patients who fulfilled the eligibility criteria were included based on consecutive sampling.

Study tool

A semi-structured questionnaire was prepared and piloted. The first part of the questionnaire collected the sociodemographic details of the participants (age, gender, occupation, residence), presence of comorbidities (diabetes mellitus (DM), hypertension, coronary artery disease), type of fistula, history of tobacco use, dialysis-related factors (nature of fistula, arteriovenous fistula flow rates, dialysis vintage, residual urine output, volume status, ultrafiltration rate per session), and biochemical values (hemoglobin, albumin, calcium, phosphorus). An electrocardiogram was done for all participants.

An echocardiogram was done for all participants and the findings were collected and recorded. Right atrial (RA), right ventricular (RV) strain pattern, RV enlargement, left ventricular (LV) strain pattern, and wall motion abnormalities were recorded. Pulmonary artery systolic pressure was calculated using the Bernoulli equation. The Bernoulli equation was used to determine estimated pulmonary artery systolic pressure (ePASP) from tricuspid regurgitant velocity (TRV) and estimated RA pressure as follows: ePASP = 4 (TRV) + RA pressure. Further, the mPAP was calculated from ePASP using the following standard formula: mPAP = 0.61 (ePASP) + 2 mmHg [9]. Other echocardiogram findings recorded were left ventricular hypertrophy (LVH), ejection fraction (EF), RV pressure, heart failure with reduced ejection fraction (HFrEF), heart failure with preserved ejection fraction (HFpEF), regional wall motion abnormality, and mitral regurgitation.

Data collection

Patients fulfilling the eligibility criteria were included in the study during the recruitment period. Before the creation of the arteriovenous fistula, an electrocardiogram and echocardiogram were done for all participants to screen for PH. Patients who had already developed PH were excluded at this stage. Proforma was administered to collect the sociodemographic details, clinical profile, and biochemical values. An arteriovenous fistula was created for the hemodialysis procedure. After three months, a repeat echocardiogram was performed for all participants. ​​​​​Initial and three-month echocardiogram findings (pulmonary artery pressures) were compared. All hemodialysis patients were dialyzed twice weekly for four hours/day with blood flow rates of 250-300 mL/minute and dialysate flow rates of 500 mL/minute using bicarbonate buffer and first-use synthetic dialyzer membranes (F6 polysulfone). Dialysis dose was determined with single-pool K (dialyzer clearance of urea) t (dialysis time), and V (volume of distribution of urea) (Kt/V), which was greater than 1.2 in each patient.

Statistical analysis

Data were collected in Microsoft Excel 2016 (Microsoft Corp., Redmond, WA, USA) and analyzed using SPSS software version 25 (IBM Corp., Armonk, NY, USA). Categorical variables were expressed as frequency and percentages, and continuous variables were expressed as mean ± standard deviation (SD). The chi-square test was used to test the association between categorical variables, and the independent sample t-test or Mann-Whitney U test was used to test the significant difference in means/mean ranks. The relationships between continuous variables such as ultrafiltration volume and pulmonary artery pressure (PAP) or mean arterial pressure (MAP) were tested using Pearson correlation analysis. Variables significant on bivariate analysis were further tested using multivariate logistic regression analysis to determine the predictors of PH. P-values <0.05 were considered statistically significant for all comparisons.

## Results

**Figure 1:**
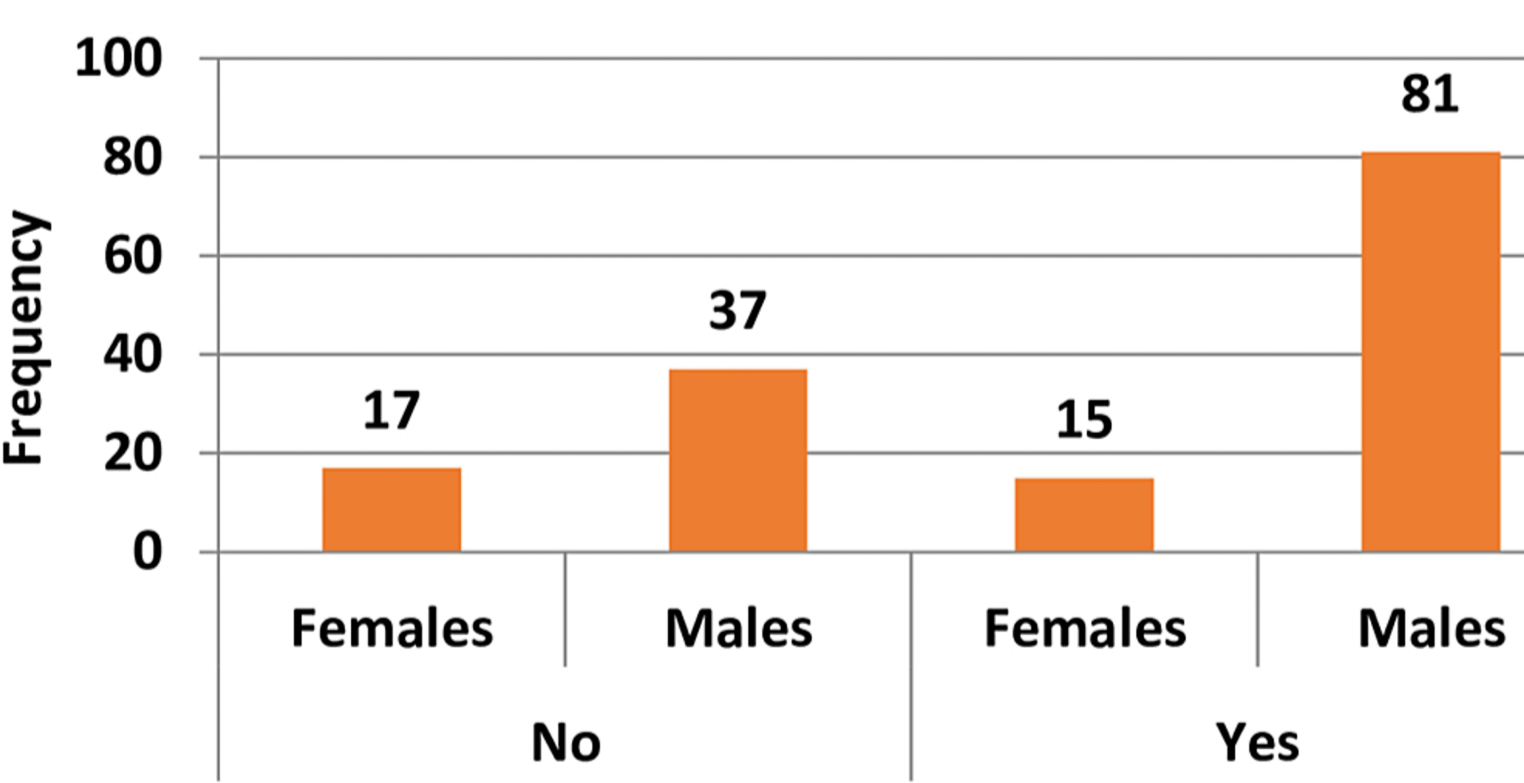
Gender distribution according to pulmonary hypertension status (n = 150).

Of the 150 study participants, 96 (64%) had PH. Among the 96 PH patients, 81 (84%) were males and 15 (16%) were females. Gender distribution according to PH status is depicted in Figure 1.

Overall, 58 (60.4%) patients were diagnosed with type 2 DM, 89 (93%) had undergone dialysis twice a week (p = 0.38), 52 (54%) were on dialysis for 6-12 months, 20 (21%) were on dialysis for 1-2 years, and 50 (52.1%) had Kt/v ≥1.2. Further, 67 (69.8%) patients had chronic glomerulonephritis, and 25 (26.04%) had chronic tubulointerstitial nephritis. The frequency of dialysis, dialysis vintage, and type of renal diseases among study participants are presented in Table 1.

**Table 1:** Dialysis frequency, vintage, and frequency of dialysis among study participants (n = 150). PH = pulmonary hypertension; CGN = chronic glomerulonephritis; CTIN = chronic tubulointerstitial nephritis

Dialysis frequency	Without PH (n = 54), N (%)	With PH (n = 96), N (%)	Total (n = 150), N (%)	Chi-square value	P-value
Thrice a week	3 (5.5)	7 (7.2)	10 (6.7)	0.745	0.38
Twice a week	51 (94.4)	89 (92.7)	140 (93.3)		
Dialysis vintage					
0-6 months	13 (24.07)	12 (12.6)	25 (16.7)	15.42	0.009
>6–12 months	12 (22.23)	52 (54.6)	64 (42.7)		
1–2 years	19 (35.2)	20 (20.8)	39 (26)		
2–3 years	3 (5.55)	2 (2.08)	5 (3.3)		
3–4 years	4 (7.40)	3 (3.1)	7 (4.7)		
>4 years	3 (5.55)	7 (7.3)	10 (6.6)		
Type of kidney disease					
CGN	5 (9.3)	67 (69.8)	72 (48)	55.53	<0.001
CTIN	31 (57.4)	25 (26)	56 (37.4)		
Not known	18 (33.3)	4 (41.6)	22 (14.6)		

Among the 96 study participants with PH, 54.6% were on dialysis for >6-12 months and 35.2% of participants without PH were on dialysis for 1-2 years. Interdialytic weight gain (mean ± SD) among patients with and without PH was 2.51 ± 0.84 and 2.68 ± 0.86, respectively. The average urea reduction ratio of participants without PH and with PH were 67.40 ± 13.54 (minimum-maximum = 35-90.23) and 60.24 ± 12.96 (minimum-maximum = 14.28 - 85), respectively. Body mass index, albumin level, presence of protein-energy malnutrition, anemia, type of catheter access, and hepatitis C status are presented in Table 2.

**Table 2:** Body mass index, protein-energy malnutrition, and type of catheter access among study participants (n = 150). PH = pulmonary hypertension

Variables	Without PH (n = 54), N (%)	With PH (n = 96), N %	Total (n = 150), N %	Chi-square value	P-value
Body mass index category (kg/m^2^)					
Underweight	13 (24)	36 (37.5)	49 (33)	7.52	0.05
Normal	32 (59.3)	37 (38.5)	69 (46)		
Overweight	4 (7.4)	16 (16.7)	20 (13)		
Pre-obese	5 (9.3)	7 (7.3)	12 (8)		
Presence of protein-energy malnutrition					
No	28 (51.8)	29 (19.3)	57 (38)	6.87	<0.009
Yes	26 (48.2)	67 (80.7)	93 (62)		
Type of access					
Brachiocephalic fistula	13 (24)	54 (56.2)	67 (45)	17.77	<0.001
Permanent catheter	0 (0)	3 (3.1)	3 (2)		
Radio cephalic Fistula	41 (76)	39 (40.7)	80 (53)		
Presence of severe anemia					
Hemoglobin <7g/dL	39 (72.2)	50 (53.2)	89 (59)	5.80	0.016
Hemoglobin ≥7g/dL	15 (27.8)	46 (46.8)	61 (41)		
Albumin					
<3.5	40 (74.1)	55 (57.3)	95 (63)	4.19	0.041
≥3.5	14 (25.9)	41 (42.7)	55 (37)		
Frequent hospitalization					
No	35 (65)	27 (28.1)	62 (41)	19.18	<0.001
Yes	19 (35)	69 (71.9)	88 (59)		
Hepatitis C status					
Negative	24 (44.4)	90 (94)	114 (76)	46.06	<0.001
Positive	30 (55.6)	6 (6)	36 (24)		

**Figure 2:**
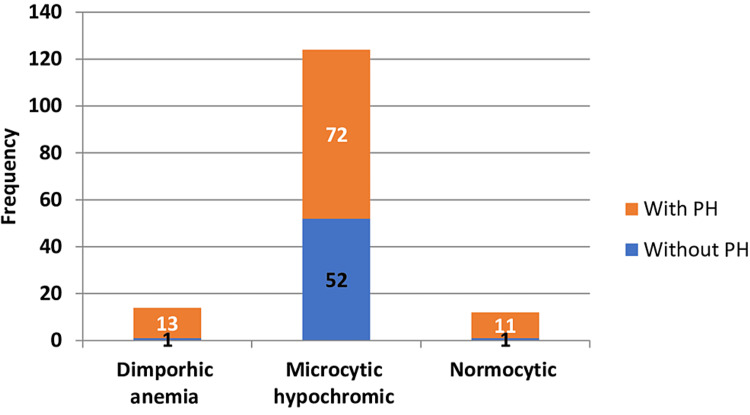
Presence of anemia among study participants (n = 150). PH = pulmonary hypertension

Of the 96 participants with PH, 80.7% experienced protein-energy malnutrition compared to 48.2% of participants without PH. The primary type of access for participants with PH was brachiocephalic arteriovenous fistula (56.2%), followed by radiocephalic arteriovenous fistula (40.7%), whereas 76% of participants without PH used radiocephalic arteriovenous fistula. Reduced albumin levels (<3.5) were found in 74.1% of participants without PH and in 57.3% of those with PH. Additionally, participants with PH had a higher frequency of hospitalizations. All these differences were statistically significant. The presence of anemia among study participants is depicted in Figure 2.

Of the 54 patients without PH, 52 (96%) had microcytic anemia. Among the participants with PH, 72 (75%) had microcytic anemia, 13 (13.5%) had dimorphic anemia, and 11 (11.4%) had normocytic anemia. The chi-square test showed a statistically significant difference (χ^2^ = 10.94, p = 0.004) between the presence of anemia in participants with and without PH. The echocardiographic findings of the study participants are presented in Table 3.

**Table 3:** Echocardiographic findings of left ventricular hypertrophy, pericardial effusion, and tricuspid regurgitation among study participants (n = 150).

Variables	Without pulmonary hypertension (n = 54)	With pulmonary hypertension (n = 96)	Chi-square value	P-value
	N (%)	N (%)		
Presence of left ventricular hypertrophy	39 (72)	88 (92)	10.06	0.002
Presence of pericardial effusion	0 (0)	62 (65)	59.44	<0.001
Presence of tricuspid regurgitation	9 (16)	96 (100)	114.28	<0.001

**Figure 3:**
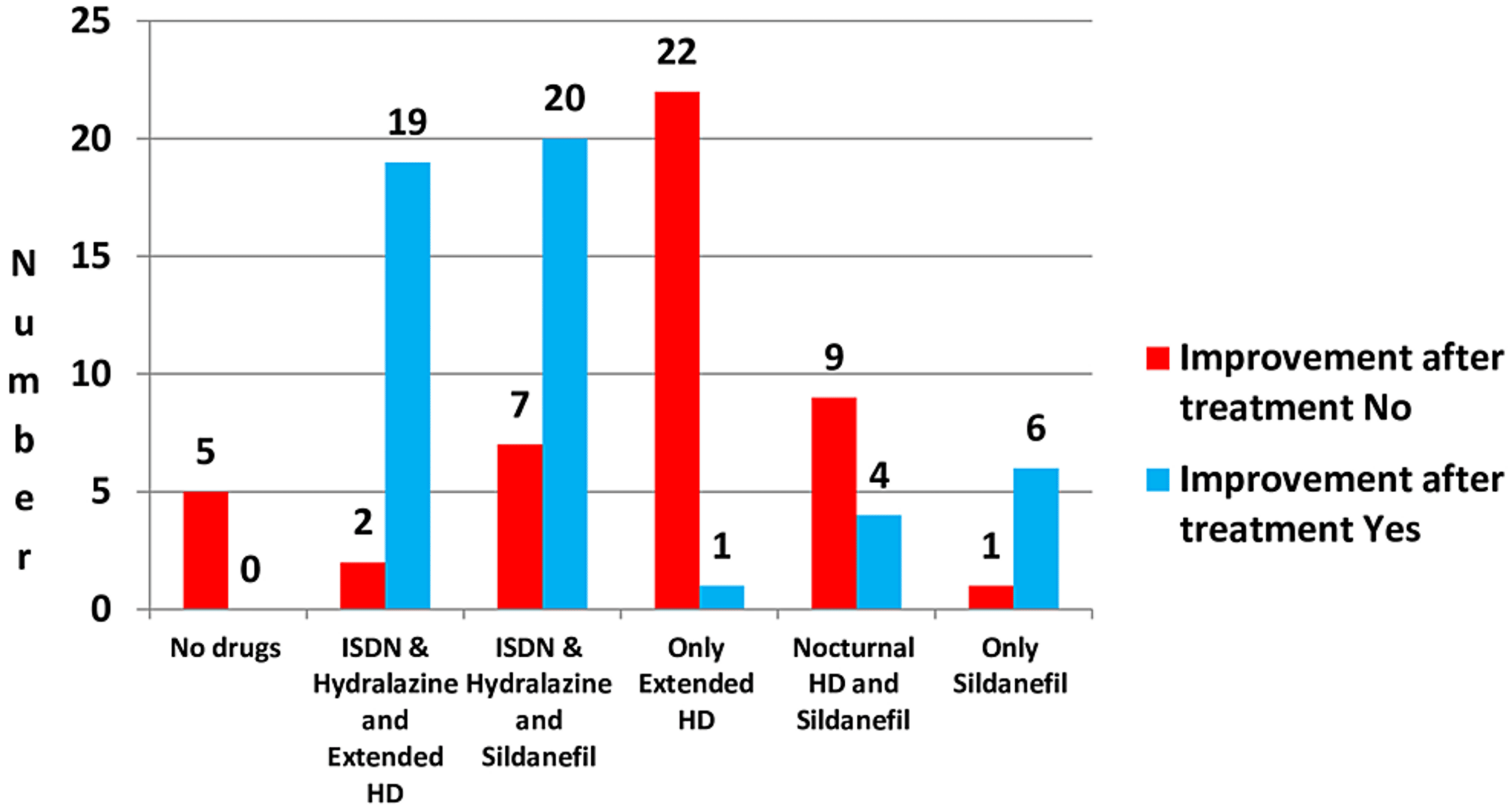
Medications administered and treatment outcomes. ISDN = isosorbide dinitrate; extended HD = extended hemodialysis; Nocturnal HD: nocturnal hemodialysis

Participants with PH exhibited more LVH, pericardial effusion, and tricuspid regurgitation on echocardiograms compared to those without PH. The ejection fraction in patients with PH was 44.54% ± 8.68%, and in patients without PH, it was 57.59% ± 4.52%. The difference was statistically significant (p = 0.001). The various medications administered and the resulting outcomes among study participants are illustrated in Figure 3.

Patients who were treated with the combination drugs isosorbide dinitrate (ISDN) and hydralazine; extended hemodialysis; and ISDN, hydralazine, and sildenafil showed significant improvement when compared with other modalities of treatment. According to drug consistency, an effect was noted in hydralazine due to its arterial dilation property, which decreases the afterload in cardiac output. Sildenafil improves pulmonary circulation through its pulmonary vasodilatory property. In this study, these drugs were found to be more effective than extended hemodialysis because patients took the drugs seven days a week, twice a day, whereas extended hemodialysis was only administered twice weekly. Thus, the total exposure to the treatment measures was higher with the drugs.

## Discussion

This study was conducted among 150 participants with CKD who underwent hemodialysis during the study period. A high prevalence (64%) of PH among CKD patients undergoing hemodialysis was observed in the study. Duration of dialysis, presence of severe anemia, protein-energy malnutrition (underweight), brachiocephalic arteriovenous fistula access, LVH, pericardial effusion, RV dysfunction, and tricuspid regurgitation had statistically significant association with the presence of PH.

Prevalence of pulmonary hypertension

Few studies have reported a similar high burden of PH among patients undergoing hemodialysis. A study by Mukhtar et al. found a 56% prevalence of PH among hemodialysis patients, more commonly in females [10]. In a study by Nagaraju et al. involving 52 hemodialysis patients, a PH prevalence of 54% was observed [3]. Interestingly, no distinctions were noted in clinical characteristics or dialysis-related factors between the PH and non-PH groups. However, when comparing hemodialysis patients with PH to those without, a significant association with higher rates of mitral regurgitation was evident (p = 0.002) in their study.

Type of fistula access and pulmonary hypertension

In this study, the primary type of access for participants with PH was brachiocephalic arteriovenous fistula access (56.2%), followed by radiocephalic arteriovenous fistula access (40.7%). Some studies suggest a potential association between arteriovenous fistula and PH, while others indicate it may not be significant. The brachiocephalic arteriovenous fistula is a proximal fistula and the flow through the brachiocephalic arteriovenous fistula is higher when compared to radiocephalic arteriovenous fistula; hence, higher venous return in brachiocephalic arteriovenous fistula can increase pulmonary artery pressure that leads to PH. For instance, in a study of 12 patients without initial PH, five developed PH after arteriovenous fistula creation [11]. Conversely, another prospective study of 20 ESRD patients showed a notable decrease in pulmonary artery pressure values following arteriovenous fistula creation [12].

Hormonal and metabolic disturbances associated with ESRD can lead to pulmonary arterial vasoconstriction and increased pulmonary vascular resistance. Pulmonary arterial pressure may also rise due to the high cardiac output necessitated by arteriovenous access, further exacerbated by common issues such as anemia and fluid overload.

Relationship between protein-energy malnutrition and chronic kidney disease

A review article by Zha and Qian showed that in individuals with CKD and ESRD, protein malnutrition is a prevalent concern, affecting a significant and growing number of patients worldwide [13]. Among the 96 participants with PH, 80.7% experienced protein-energy malnutrition compared to 48.2% of participants without PH in this study. This nutritional deficiency significantly increases the risk of illness and mortality. However, it was suggested that with consistent dietary monitoring and specific nutritional interventions, protein malnutrition can be effectively managed and potentially reversed. Guidelines recommend personalized protein intake strategies, suggesting approximately 1.2 g/kg/day for dialysis CKD patients.

Left ventricular hypertrophy, pericardial effusion, and tricuspid regurgitation in chronic kidney disease patients

This study showed the presence of LVH, pericardial effusion, and tricuspid regurgitation in patients undergoing hemodialysis. This can be caused by a combination of factors related to both the underlying kidney disease and the dialysis treatment itself. Chronic fluid retention in patients with ESRD often leads to fluid overload, increasing the workload on the heart, causing LVH, and, eventually, LV failure. Persistent hypertension due to CKD increases afterload (the resistance the heart must pump against), causing LV hypertrophy and stiffening, reducing its efficiency and leading to heart failure. Dialysis-related ischemia from episodes of hypotension can further reduce coronary perfusion, worsening ischemic heart disease.

This study also showed a high burden of anemia among the participants. Anemia, common in CKD due to reduced erythropoietin production, decreases oxygen delivery to the myocardium, leading to LVH and failure as the heart attempts to compensate. These factors collectively underscore the complex interplay between kidney disease and heart function, necessitating careful management and coordination of care for patients undergoing hemodialysis.

Studies have shown that subclinical LV dysfunction is common in individuals with advanced CKD and may not always be detected through standard echocardiographic evaluations [14]. Two extensive echocardiographic studies have shown that patients with PH have significantly higher left-sided filling pressures, including increased pulmonary capillary wedge pressure and larger left atrial size and these results suggest chronic volume overload in this patient group [15,16].

A study showed that among hemodialysis ESRD and CKD patients, the prevalence of pericardial effusion was 14.3%. Bentata et al. reported that the incidence of small, moderate, and large pericardial effusion among ESRD patients was 31.2%, 37.6%, and 31.2%, respectively [17]. Pericardial effusion is more prevalent in patients with CKD undergoing hemodialysis compared to other populations. Among hemodialysis patients, CKD with pericardial effusion is associated with higher mortality rates compared to those without this complication.

The combination of ISDN and hydralazine with sildenafil demonstrated superior outcomes compared to other treatment modalities, likely due to the consistent arterial and pulmonary vasodilatory effects of the drugs.

Limitations

The limited sample size may not be representative of the broader CKD population in India, limiting the generalizability of the findings. The study was conducted in a single tertiary care hospital, which may have specific patient demographics and clinical practices not representative of other settings. The cross-sectional design only provided a snapshot in time and cannot establish causality between CKD, PH, and associated factors. Longitudinal studies are required to understand the progression and potential causative factors of PH in CKD patients. The study population was limited to those registered for hemodialysis, potentially excluding CKD patients not on dialysis or those on different dialysis modalities, which may influence the prevalence and characteristics of PH. The study did not account for all potential confounding factors that could influence the association between CKD and PH, such as comorbid conditions, medication use, and lifestyle factors. The assessment of treatment efficacy was based on a relatively short follow-up period, limiting the ability to evaluate the long-term outcomes and side effects of the treatment regimens.

## Conclusions

The high prevalence of PH among CKD patients undergoing hemodialysis underscores the importance of vigilant monitoring and targeted interventions in this vulnerable population. Compared to extended dialysis, the combination of drugs has a superior effect because the drug’s effect is persistent seven days a week, whereas dialysis is used only for 12 hours a week. A drug with vasodilatory properties shows a better response in patients with PH. Additionally, correcting protein-energy malnutrition along with adequate hemodialysis will improve PH.
